# Assessment of oral health status and quality of life in hearing-impaired children from Syria

**DOI:** 10.1038/s41405-024-00242-3

**Published:** 2024-07-07

**Authors:** Alemar Nazeeh Ghannam, Mayssoon Dashash, Louei Darjazini Nahhas

**Affiliations:** 1 Damascus University, Damascus, Syria; 2 Surgery Division of Otorhinolaryngology department, Faculty of Medicine, Syrian Private University, Damascus, Syria

**Keywords:** Special care dentistry, Gingivitis

## Abstract

**Aim:**

This study aimed to evaluate the oral health status of children with hearing impairments and assess the relationship between various oral health factors and the Pediatric Oral Health-Related Quality of Life.

**Materials and methods:**

This observational cohort study involved 90 hearing-impaired children aged 6–12 years. Diagnostic tools such as pure-tone audiometry were used to evaluate their hearing abilities. Dental health was assessed by calculating DMFT, dmft, Plaque and Gingival indices. The oral health-related quality of life was measured using the POHRQoL.

**Results:**

The severity of hearing impairment varied with 3% having severe hearing loss, 13% having profound hearing loss, and 83% having complete hearing loss. A high prevalence of dental cavities with 93% of children affected was found. DMFT score was similar between males and females (2.5 ± 0.3 vs. 2.3 ± 0.3 respectively). Males exhibited a higher mean dmft score (4.1 ± 0.5 vs. 3.3 ± 0.5), and a higher mean Plaque Index (1.9 ± 0.1 vs. 1.5 ± 0.1). On the other hand, females showed a higher mean Gingival Index (0.9 ± 0.1 vs. 1.1 ± 0.2). Results indicated a decreased OHRQoL with a mean POQL score of 6.4 ± 2.89. Spearman’s test revealed a significant positive correlation between POQL total score and DMFT scores (*P* = 0.000), dmft scores (*P* = 0.000), Plaque Index scores (*P* = 0.000), and Gingival Index scores (*P* = 0.038). There was a weak positive correlation between hearing impairment severity and POQL total score, it was not statistically significant.

**Conclusions:**

Children who have hearing impairments exhibit poor oral health, and levels of dental caries, which can negatively impact their overall POHRQoL. Therefore, it is highly recommended to start specialized oral health education and comprehensive dental care programs to improve their OHRQoL.

## Introduction

The term “hearing impairment” encompasses a range of decreased hearing abilities, from minimal impairment to complete deafness, which can be inherited or acquired over time due to disease processes [1]. Approximately 430 million people worldwide require treatment for their hearing impairment, with over 1.5 billion affected globally, and 80% living in low- and middle-income countries [2]. In Arab countries, hereditary hearing loss rates vary, ranging from 1.20 to 18 per 1000 births annually [3]. Syria highlights the importance of school-based screening for hearing impairment prevalence in school-age children [4].

Oral health significantly affects speech, chewing, and swallowing, crucial for nutrition, communication, confidence, and well-being, particularly for individuals with special needs [5]. According to a study conducted in Nigeria, it was found that individuals with hearing impairments have a higher incidence of dental caries compared to their counterparts with visual and physical impairments [6]. Additionally, they exhibit poor periodontal health as was found in a study conducted in India [7]. Challenges in mastering oral hygiene skills and accessing qualified dentists contribute to poor oral health status among children with hearing impairments [8].

The concept of Oral Health-related Quality of Life (OHRQoL) assesses the functional, social, and psychological effects of oral diseases [9], revealing significant differences between healthy children and those with general diseases [10]. Mild to moderate hearing impairment can lead to negative consequences affecting psychological, behavioral, social, and emotional development [11], impacting oral hygiene practices and quality of life [12].

Globally, there is an emphasis on measuring quality of life to influence healthcare decisions effectively [13]. Pediatric Oral Health-related Quality of Life (POHRQoL) encompasses physical, psychological, and social aspects affected by oral health conditions [14].

Despite the increasing levels of hearing loss and poor oral conditions, there is insufficient data on oral health outcomes among children with hearing problems in Syria.

Identifying oral health status and OHRQoL among children with hearing impairments is crucial for improving their oral health outcomes and reinforcing the evidence base for oral health policies. Therefore, the main objective of the study was to determine oral health status among hearing-impaired children and assess the relationship between oral health factors and POHRQoL among children aged 6-12 years.

## Materials and methods

### Ethical considerations

Ethical approval was obtained from the Directorate of Social Affairs and Labor in Damascus, and the study protocol was approved by the Scientific Research and Postgraduate Board of Damascus University, Ethics Committee reference number (IRB No. UDDS-2649-02082021/SRC-1550).

Additionally, parental consent was obtained for their children’s participation after informing them about the research process and objectives. The examiner had experience in examining/treating children and assured that they were comfortable with the examination.

### Study population and inclusion criteria

During the academic year 2022, the Institute of Special Education for the Hearing Impaired accommodated 110 children aged 6–12 years with hearing impairment. This study opts for a total population approach rather than sampling resulting in the inclusion of 90 participants with hearing impairment, after excluding 20 children. The study’s sampling frame was a close representation of the target population.

All participants in the study were selected based on the approval of their parents through a signed informed consent. Eligible participants were hearing-impaired children of Syrian descent aged 6 to 12 years meeting the study’s criteria. Exclusions were based on the presence of other systemic diseases, prolonged school absenteeism, undergoing orthodontic treatment, or uncooperativeness.

Participants were required to comprehend information provided by classroom teachers in sign language.

### Study protocol and data collection

The assessment included pure-tone audiometry for hearing-impaired children [15] to classify the severity of hearing impairment, severity was classified as per recommendations by the Global Burden of Disease Expert Group on Hearing Loss [16].

An oral visual examination was performed by the principal investigator, using appropriate equipment to investigate caries, plaque accumulation, and gingivitis. Personal information, DMFT and dmft indices [17], Silness-Löe Plaque Index (PI), and Gingival Index (by Löe and Silness) (GI) [18] were recorded.

The children were seated, using a head-held light, disposable gloves, dental mirror, and probes to investigate caries. Tongue depressors and cotton rolls were used to remove food residues and moisture that could obstruct the direct vision of the teeth.

The oral health-related quality of life of these children was assessed using the Pediatric Oral Health-Related Quality of Life (POQL) [14], through a pilot study (*n* = 10) followed by the main assessment to ensure its suitability and applicability. Which included 4 (36.4%) males and 7 (63.6%) females, with hearing impairments, aged 6–12 years. The reliability of the POQL questionnaire was confirmed by Cronbach’s alpha test, showing a reliability of 0.82. The results of the pilot study showed that 9 (90%) were avoiding smiling or laughing, 8 (80%) were feeling worried about their attractiveness, 6 (60%) were unhappy with the appearance, 5 (50%) had difficulties in paying attention, 4 (40%) were missing school, 6 (60%) had pain, 5 (50%) were unable to eat certain food (hard, hot, or cold), 4(40%) were feeling anger 4 (40%), 7 (70) were worry, and 8 (80%) were crying. Consequently, no modification was required. Children were instructed to carefully read and understand each question and select the answer that best described their experience over the last three months. Additionally, a full explanation of the questionnaire was provided to teachers and sign language interpreters in order to provide support to each child individually if required.

### Statistical analysis

Statistical analysis included descriptive statistics. Cronbach’s alpha test for assessing the validity and reliability of the questionnaire, and Spearman’s test to determine the correlation between variables. Data analysis was performed using Statistical Software Package SPSS version 25.0 with a significance level set at 5% (*P* < 0.05).

## Results

Ninety children between the ages of 6 and 12 years with hearing impairment were included. Among these, 27 (30%) were aged 6–8 years, and 63 (70%) were aged 9–12 years. The gender distribution showed that 58 (64%) were males, and 32 (36%) were females. The mean was 9.7 ± 0.25 for males, and 9.6 ± 0.31 for females. The severity of hearing impairment varied among the children: 3% had 3% severe hearing loss (65–79.9 dB), 13% had profound hearing loss (80–94.9 dB), and 83% had complete hearing loss (≤ 95 dB). Female participants exhibited more severe impairment compared to males (mean 2.9 ± 1.93 vs. 1 ± 0.01 respectively).

The findings revealed that 84 out of 90 children (93%) had dental caries, while 6 (7%) did not. In terms of permanent teeth, the average DMFT was similar for males and females (2.5 ± 0.25 vs. 2.3 ± 0.33 respectively).

However, males had a higher mean dmft score than females (4.1 ± 0.49 vs. 3.3 ± 0.52). Males also had a higher Plaque Index (PI) score, indicating more plaque buildup (1.9 ± 0.81 vs. 1.5 ± 0.09 for females), whereas females had a higher mean Gingival Index (GI), suggesting more gingivitis compared to males (0.9 ± 0.09 vs. 1.1 ± 0.14). Findings are presented in Table 1.

**Table 1:** Descriptive statistics of the sample.

Variables		Male		Female	
		Mean	SD^a^	Mean	SD^a^
Age		9.7	0.25	9.6	0.31
HI		1	0.01	2.9	1.93
Dental Indices	DMFT	2.5	0.25	2.3	0.33
	dmft	4.1	0.49	3.3	0.52
	PI	1.9	0.81	1.5	0.09
	GI	0.9	0.09	1.1	0.14

*M* male, *F* female. *HI* Hearing Impairment, *DMFT* decay, missing and filling score (permanent teeth), *dmft* decay, missing and filling score (primary teeth), *PI* plaque index, *GI* gingival index.

^a^*SD* standard deviation.

The validity and reliability of the POQL questionnaire were confirmed by Cronbach’s alpha test, showing a reliability of 0.76 and validity of 0.82 (Table 2).

**Table 2:** Cronbach’s Alpha test for assessing the validity and reliability of the Pediatric Oral Health-Related Quality of Life questionnaire.

Number of Items	Reliability index	Validity index
10	0.76	0.82

The responses to individual items in the POQL questionnaire showed significant diversity among study subjects. A notable percentage expressed specific concerns, with a majority reporting avoiding smiling or laughing 65 (72.2%), feeling worried about their attractiveness 60 (66.7%), being unhappy with appearance 56 (62.2%). Additionally, a significant portion reported experiencing challenges such as paying attention 54(60%), missing school 46 (51.1%), pain 69 (50.7%), inability to eat certain food (hard, hot, or cold) 61 (67.8%), worry 50 (55.6%), and crying 76(84.4%). Conversely, fewer participants reported feeling anger 30 (33.3%). The study participants reported a POQL score of 6.4 ± 2.89. See Table 3 for details.

**Table 3:** Descriptive analysis for the Pediatric Oral Health-related Quality of Life questionnaire.

Questions	Answers	Number (Percent)	Mean ± SD^a^
1. Not smile/laugh	No	25 (27.8%)	0.7 ± 0.45
	Yes	65 (72.2%)	
2. Worry less attractive	No	30 (33.3%)	0.7 ± 0.47
	Yes	60 (66.7%)	
3. Unhappy with looks	No	34 (37.8%)	0.6 ± 0.49
	Yes	56 (62.2%)	
4. Pay attention	No	36 (40%)	0.6 ± 0.49
	Yes	54 (60%)	
5. Miss school	No	44 (48.9%)	0.5 ± 0.50
	Yes	46 (51.1%)	
6. Pain	No	21 (23.3%)	0.8 ± 0.43
	Yes	69 (76.7%)	
7. Eat food (hard/hot/cold)	No	29 (32.2%)	0.7 ± 0.47
	Yes	61 (67.8%)	
8. Angry	No	60 (66.7%)	0.3 ± 0.47
	Yes	30 (33.3%)	
9. Worry	No	40 (44.5%)	0.7 ± 1.2
	Yes	50 (55.6%)	
10. Cry	No	14 (15.6%)	0.8 ± 0.4
	Yes	76 (84.4%)	

^a^Standard deviation.

Spearman’s test identified a significant positive correlation between the total score of POQL and the following factors: DMFT scores (*r* = 0.476, *P* = 0.000), dmft scores (*r* = 0.584, *P* = 0.000), Plaque Index (PI) scores (*r* = 0.637, *P* = 0.000), as well as Gingival Index (GI) scores (*r* = 0.220, *P* = 0.038). Conversely, the severity of hearing impairment showed a weak positive correlation with POQL total score, although this relationship did not reach any statistical significance (Table 4).

**Table 4:** Relationship between POQL score and both dental indices and hearing impairment.

	POQL score	
	Correlation coefficient	Sig. (2-tailed)
DMFT	0.476^a^	**0.000**
Dmft	0.584^a^	**0.000**
Plaque Index	0.637^a^	**0.000**
Gingival Index	0.220^b^	**0.015**
Hearing Impairment	0.116	0.278

^a^Correlation is significant at the 0.01 level.

^b^Correlation is significant at the 0.05 level.

Significance of bold values, *p* < 0.05.

## Discussion

The evaluation of OHRQoL can help identify oral health issues that impact children on a day-to-day basis [19]. However, there is a scarcity of information regarding this aspect specifically concerning children with hearing impairment [20]. Selecting this particular age group is justified by the fact that children are particularly sensitive during their formative years, posing unique challenges in maintaining good oral health. Poor oral health in childhood can also serve as an indicator of potential risks later in life [21].

The study’s findings revealed significantly elevated rates of dental caries among hearing-impaired children, aligning with the findings of Azizah et al, who reported a high prevalence of dental caries (64.3%) [22]. This trend may be influenced by factors such as age, the severity of hearing loss, living conditions, and the level of support received from parents, siblings, or caregivers for oral hygiene, many of whom may lack sufficient education or knowledge about oral hygiene and appropriate nutrition for children with disabilities [23].

Moreover, the present study identified issues of poor oral health including plaque accumulation and gingivitis in children with hearing impairment, consistent with the findings of a study by Jnaneswar et al. [7]. This could be due to inadequate oral hygiene practices, a lack of commitment to oral care, unhealthy dietary habits, and limited awareness among parents, leading to delays in seeking dental treatments. However, the correlation between caries and hearing impairment merits further study, as qualitative research could be useful in exploring the reasons for certain correlations. Additionally, children with hearing impairment demonstrated suboptimal performance in areas such as postural control, motor skills, and overall quality of life [24]. On the contrary, Rajabloo et al. reported that the oral health status of children with hearing impairments was rated by parents as very good or good at a rate of 57.4%. This could be due to the participants attending specialized schools with perfect organization and attention to oral hygiene behaviors [25].

OHRQoL questionnaires provide a patient-centered assessment of oral health, taking into account the various dimensions of health and the functional and psychosocial impacts of dental conditions [26]. Numerous studies have been conducted at Damascus University focusing on assessing oral health-related quality of life among children with systemic diseases, such as those with cleft lip and palate [27], children with heart diseases [10], and others. However, there has been no investigation into this matter among children with hearing impairments in Syria.

POHRQoL was used, in this study, due to its high validity, reliability, and suitability for self-administration. It offers a convenient way to assess oral health, and it is appropriate for participants aged between 6-12 years [14]. Hearing-impaired children completed the questionnaire with the assistance of teachers and sign language interpreters, facilitating effective communication during the assessment of the POHRQoL items.

The investigation into POHRQoL revealed a reduced quality of life among hearing-impaired children, aligning with the findings by Manohar & Subramaniam, as well as Singh et al. studies [20, 28].

In our study, item 10 (cry) was noted as the most commonly affected performance due to poor oral health, according to the POHRQoL results. In contrast, Rahman et al. found that the most prevalent oral health problem among HI children was sensitive teeth, using the impact of oral health on daily living (Child-OIDP) tool [29], while a study by Alkahtani highlighted that the inability to speak clearly was the most affected performance due to poor oral health, as demonstrated by the GOHAI-Ar inventory [30].

The correlation between poor oral health and low oral health-related quality of life in hearing-impaired individuals highlights the need for a nuanced exploration of interconnected factors. Firstly, communication barriers pose a significant challenge. Managing a dental appointment without clear communication, understanding instructions, expressing concerns, or comprehending treatment options, becomes challenging. This breakdown in communication can result in misunderstandings, inadequate treatment, and increased frustration, ultimately negatively impacting the child’s quality of life [31].

Consequently, untreated dental issues may escalate, worsening oral health problems and reducing overall well-being [32].

Moreover, traditional methods may not adequately cater to the needs of hearing-impaired children when it comes to understanding oral hygiene instructions or recognizing oral health indicators [33]. It may be attributed to reports indicating that children with hearing impairments are observed to have more mental health issues and communication problems than typically hearing children [34].

The social and psychological consequences should not be underestimated. Individuals with hearing impairment may encounter social isolation, stigma, and psychological distress, intensifying their oral health concerns, as reported by Patel et al. [35, 36]. These factors can impact attitudes towards oral care and willingness to seek treatment, perpetuating the cycle of poor oral health and diminished quality of life.

The study revealed a significant positive correlation between POHRQoL and DMFT, dmft, PI, and GI scores, indicating that enhanced oral health status correlates with an enhanced quality of life in hearing-impaired children. Additionally, a weak positive correlation exists between hearing impairment and POHRQoL, suggesting a somewhat favorable OHRQoL. In contrast to the study by Alkahtani et al. [30], which found that the clinical oral indices such as high OHI-S, PI, decayed teeth, missing teeth, and DMFT scores all have shown negative correlation with GOHAI-Ar.

This underscores the importance of maintaining good oral hygiene practices and promptly addressing dental concerns in hearing-impaired children to enhance their overall well-being. These findings emphasize the necessity for tailored and comprehensive oral health interventions designed to meet the specific needs of this demographic, ultimately enhancing their quality of life.

During the study involving children with hearing impairments, several limitations were identified. These include the absence of a dedicated questionnaire for gathering information on OHRQoL. Moreover, no data on the participants’ socioeconomic status were gathered in relation to POHRQoL. Future studies should consider obtaining a larger sample size from different regions of Syria to confirm these findings.

## Conclusion

Children who have hearing impairments exhibit poor oral health, and levels of dental caries, which can negatively impact their overall POHRQoL. Therefore, it is highly recommended to start specialized oral health education and comprehensive dental care programs to improve their OHRQoL.

## Data Availability

The datasets used and/or analyzed during the current study are available from the corresponding author on reasonable request.
