# Correction: Assessment of oral health status and quality of life in hearing-impaired children from Syria

**DOI:** 10.1038/s41405-024-00250-3

**Published:** 2024-08-05

**Authors:** Alemar Nazeeh Ghannam, Mayssoon Dashash, Louei Darjazini Nahhas

**Affiliations:** 1 Damascus University, Damascus, Syria; 2 Surgery Division of Otorhinolaryngology Department, Faculty of Medicine, Syrian Private University, Damascus, Syria

Correction to: *BDJ Open* 10.1038/s41405-024-00242-3, published online 07 July 2024

When originally published on 7 July 2024, the author list order was incorrect. Please see the correct author list order below:

Alemar Nazeeh Ghannam,^1,*^ Mayssoon Dashash^1^ and Louei Darjazini Nahhas^2^

^1^Damascus University, Damascus, Syria. ^2^Surgery Division of Otorhinolaryngology Department, Faculty of Medicine, Syrian Private University, Damascus, Syria.

^*^email: Alemar.ghannam.2222@hotmail.com

The authors apologise for any inconvenience caused.

